# Mitigating the Impact of Admixtures in Thai Herbal Products

**DOI:** 10.3389/fphar.2019.01205

**Published:** 2019-10-15

**Authors:** Santhosh Kumar J. Urumarudappa, Chayapol Tungphatthong, Suchada Sukrong

**Affiliations:** Research Unit of DNA Barcoding of Thai Medicinal Plants, Department of Pharmacognosy and Pharmaceutical Botany, Faculty of Pharmaceutical Sciences, Chulalongkorn University, Bangkok, Thailand

**Keywords:** admixtures, adulterations, medicinal plants, Thai herbal products, herbal database

## Abstract

Medicinal plants and their products are extensively used within indigenous healthcare systems in Thailand and several other nations. The international trade of herbal products has a noteworthy impact on the worldwide economy, and the interest in herbal products is expanding in both developing and developed countries. There has been rapid growth in the medicinal plant product market and a broadening consumer base interested in herbal products from Thailand. However, in herbal industries, ingredient substitution and admixture are typical issues wherein species of lower market value are admixed with those of a higher value. The adverse consequences of consuming adulterated drugs are invariably due to the presence of an unintended herb rather than the presence of an intended herb. It has also been argued that admixtures are intentional because of the lack of regulatory policies or centralized tests for product authentication. The consequences of species admixtures can extend from the reduced efficacy of a drug to decreased trade value. This study aims to clarify the nature and extent of species admixtures reported in the Thai herbal trade market and discuss the potential reasons for such adulteration. In the broader context of species admixtures, we strongly propose the establishment of multiple herbal crude drug repositories that can be developed to facilitate the use of comparative identity tests by industry, traders, and researchers to maintain authentic natural health product (NHP) standards and to certify the authenticity of NHPs. The proposition of the establishment of centralized testing (CT) could be a promising initiative in Thailand for the development of science and technology, and the herbal medicines produced as a result of CT could be dispensed as prescription drugs based on disease consideration instead of as health foods or nutraceuticals.

## Introduction

Medicinal plants contribute significantly to both the indigenous and modern systems of medicine. Since ancient times, medicinal plants have been used in traditional home remedies and have formed a major component of the indigenous system of medicine practiced in many countries. It has been reported that 80% of the world’s population depend on traditional herbal medicines, mainly plant-based herbal drugs, for their primary healthcare (Srirama et al., 2017). The international trade of medicinal plants and their herbal products contributes to the worldwide economy; thus, the demand for such herbal products is growing in both developing and developed countries. More than 28,187 species of plants are used for medicinal purposes (Medicinal Plant Names Services Portal, 2017). These plants are used as either herbal medicines by the indigenous and rural communities or in the manufacture of pharmaceutical drugs. Currently, there are more than 1,000 herbal companies producing medicinal plant products, and their annual income is in excess of $60 billion (Newmaster et al., 2013). Due to commercialization and increased demand for traditional herbs, safety, quality, and assurance are major issues (Chen et al., 2017).

In recent years, an international resurgence of attention has occurred in the use of herbal products in human healthcare, which has led the herbal product market to thrive (Barry and Abratt, 2015). The increase in demand has far outpaced the availability of raw materials. Thailand’s major exports of medicinal plants are used in pharmaceutical, perfumery, etc. Estimated exports of herbal products from Thailand indicate an increase from US$1.4 million in 2007 to US$11.5 million in 2011, recording a compounded annual growth rate of 12.67%. The major export markets of Thai herbal extracts are Myanmar (38.72%), the United States (13.15%), Cambodia (10.92%), Japan (9.50%), the United Kingdom (6.79%), and Hong Kong (3.31%), whereas the import markets include China, Brazil, the United States, Germany, Philippines, and India (https://report.nat.gov.tw). However, due to high demands, the substitution and adulteration of many medicinal plant species in medicinal plant products (Techen et al., 2004; Song et al., 2009; Suwanchaikasem et al., 2013; Urumarudappa et al., 2016; Srirama et al., 2017; Dechbumroong et al., 2018; Kumar et al., 2018), breweries such as teas (Stoeckle et al., 2011), and “nutraceuticals” (Bruni et al., 2010; Jaakola et al., 2010) have been documented.

Medicinal plants used in raw herbal trade are often marketed as powders; dry twigs including dry roots, rhizomes, leaves, etc.; and pills or tablets, and thus are usually difficult to identify morphologically. The major problems with the trade of raw herbal products include the adulterations and substitution of certain species with morphologically similar and geographically co-occurring species (Ved and Goraya, 2007; Urumarudappa et al., 2016; Srirama et al., 2017). In most of the Southeast Asian countries, including Thailand, over 80% of the medicinal plants that are in active trade are seldom cultivated. These are collected largely from the wild. The collection of these medicinal plants is largely performed by local people who often rely only on their knowledge or experience in identifying the species being collected (Menon, 2003; Seethapathy et al., 2015; Srirama et al., 2017). Thus, most often, there are admixtures of related species or morphologically similar species in the herbal trade samples. The possible reasons for adulteration are often attributed to the apparent confusion associated with local or vernacular names of plant species, lack of accurate raw plant material, and identical morphological characters of co-occurring species of plants (Mitra and Kannan, 2007; Kumar et al., 2018). Admixture is a common malpractice in raw material trade and a major problem in the herbal industry. Admixture is the practice of substituting an authentic crude drug partially or entirely with a different drug similar to the original drug but inferior in therapeutic and synthetic properties or the addition of low-efficiency drugs for the intentional or unintentional enrichment of profits (Mukherjee, 2002; Kokate et al., 2007). The adverse consequences of consuming adulterated drugs are invariably due to the presence of an unintended herb rather than the presence of an intended herb (Sunita, 1992; Uniyal and Joshi, 1993; Sarin, 1996). It has also been argued that admixture is deliberate because there is no standard monitoring tool in place or commercial test for product authentication. The consequences of species admixtures can extend from the reduced efficiency of a drug to the decreased trade value (Wieniawski, 2001), in addition to a threat to the safety of consumers (Song et al., 2009).

Since adulteration is very difficult to trace *in situ* and requires expertise, scientific methods have been developed to address medicinal plant adulteration in herbal trade, such as microscopic, macroscopic, organoleptic, and chemical analyses (Techen et al., 2014; Moraes et al., 2015; Mishra et al., 2016; Pawar et al., 2017; Raclariu et al., 2018). DNA technology has been heavily used to investigate the adulteration of species (Newmaster et al., 2013; Phoolcharoen and Sukrong, 2013; Kumar et al., 2015; Urumarudappa et al., 2016; Pawar et al., 2017; Dechbumroong et al., 2018; Liu et al., 2018; Raclariu et al., 2018; Seethapathy et al., 2018; Speranskaya et al., 2018; Kreuzer et al., 2019). Among the available tools for species identification and traceability, DNA barcoding is a low-cost and highly efficient approach that has the potential for automatization and rapid application (Galimberti et al., 2013; Mishra et al., 2016; Dechbumroong et al., 2018).

In this review, we attempt to elucidate the nature and extent of species adulteration reported in the Thai herbal trade market and discuss the possible underlying causes of adulteration. In the larger context of species admixtures, we believe and propose the need to certify the centralized system to develop an efficient mechanism to systematically evaluate traditional medicine and connect it with both national and international trade regulators. We strongly suggest the establishment of several crude herbal drug depositories to maintain authentic biological reference materials (BRMs) that can be developed to facilitate the use of comparative identity tests by industry, traders, and researchers and to certify herbal product authenticity. We strongly recommend the enforcement of existing laws and regulations concerning the quality of herbal products to govern herbal trade around the globe. The proposed establishment of centralized testing (CT) could be a promising initiative in Thailand for the development of science and technology. The herbal medicines produced as a result of CT could be dispensed as prescription drugs based on disease consideration instead of being designated health foods or nutraceuticals. CT could pave the way to increase the country’s market size of phytomedicine and become a novel strategy in Thailand as an important contributor to the herbal product market and to maintain the safety and health of consumers.

## Materials and Methods

### Literature Search on Medicinal Plants Used in Thai Herbal Pharmacopoeia (THP)

In this study, the list of plants in Thailand Herbal Pharmacopoeia (THP) was the main focus. A literature search was performed with various electronic databases [Google Scholar, Science Direct (Scopus), and Web of Science] using specific search terms such as “Thai Herbal Pharmacopoeia mentioned plant names,” “Thai medicinal plants,” “Admixtures of Thai medicinal plants,” and “Thai herbal medicine,” which included peer-reviewed scientific studies and reports used to document traditional medicinal plants used in THP.

### Origin of Thai Traditional Medicine (TTM)

Traditional medicines have been established after extensive experiences of over 100 years of practicing physicians in the indigenous systems of medicine (Kamboj, 2000). Traditional medicine of Thailand has an extensive history of use in various treatments and illnesses since the Sukhothai period (1238–1337) of Thai history. By the knowledge passed down through generations, a well-defined structure of traditional medicine has been developed, systematized, and is now recognized as Thai Traditional Medicine (TTM) by the Thailand Kingdom (SEARO, WHO, 2010). TTM is considered holistic medicine, as it involves not only the use and production of herbal medicine but also diagnosis, treatment, and pharmacy practice (Akarasereenont et al., 2015). The famous Thai massage (Nuad Thai) also originates from this system.

TTM draws its traditional content from the Indian and Chinese systems (WHO, 2002), as indicated by the diversity of ethnic groups in Thailand’s population (Trisonthi and Trisonthi, 1995), possibly due to historic migration from neighboring countries (Phumthum and Balslev, 2018). Maintenance of unique and distinctive knowledge of medicinal plants has been seen mainly in these communities. Additionally, a study on a typical mountain-residing indigenous community named “Karen” showed that having limited access to public healthcare systems encouraged them to accumulate a valuable method of preventing and treating diseases with herbal remedies (Tangjitman et al., 2015). However, TTM observed a decline in consumer acceptance from 1916 to 1977 due to the influence of Western medicine, although a subsequent revival in acceptance was observed after the WHO advised that traditional medicine and plant herbal medicine should be implemented in primary healthcare systems (Akarasereenont et al., 2015). Then, to accelerate the integration of traditional medicine into national healthcare system, a list of the national essential herbal drugs (NEHD) was established in 1999 according to the National Drug Committee (2006). Today, the Thai government also funds and strongly supports medicinal plant research and development.

To reduce the cost of imported drugs and improve access to healthcare, ASEAN members agreed to implement the WHO’s traditional medicine strategy (WHO, 2002) in the year 2004, which in turn enhanced the use of traditional medicine, especially herbal medicine, in ASEAN healthcare systems. A recent investigation reports that Thai people spend several million bahts *per* year on unproven herbal products (Osathanunkul et al., 2016). Currently, alternative medicines are popular not only in developing countries, such as Thailand and India, but also in developed countries, such as the United States, Canada, and Europe. In support of this, an estimate shows that the United States alone spends o ver US$5 billion *per* year on herbal products (Osathanunkul et al., 2016). However, herbal products are considered supplements rather than medicines in the United States, which may be due to the absence of clinical data on their safety and efficacy.

### Species Admixtures in Herbal Trade: Thai Herbal Pharmacopoeia

THP has listed 66 medicinal plants with a high therapeutic index as herbal medicine (**Table 1**). This study considered the listed medicinal plants to understand their status under the threat of adulteration in herbal trade (**Table 2**). A previous report has shown that 15.5% of the total number of plant species in Thailand is used as medicinal plants (Wakdikar, 2004). Recently, a number of studies have also shown that natural health product (NHP) market samples of raw herbal trade materials are often adulterated with other species (Kool et al., 2012; Newmaster et al., 2013; de Boer et al., 2015; Kumar et al., 2015; Urumarudappa et al., 2016; Mezzasalma et al., 2017; Pawar et al., 2017; Dechbumroong et al., 2018; Raclariu et al., 2018; Liu et al., 2018; Seethapathy et al., 2018; Speranskaya et al., 2018; Tungphatthong et al., 2018; Kreuzer et al., 2019).

**Table 1:** Information on the 66 documented medicinal plants used in the Thailand Herbal Pharmacopoeia 2018.

Thai name	Scientific name	Family	Habit	Parts used	Treatment/application
Wannam (วานน้ำ)	*Acorus calamus* L.	Acoraceae	Aquatic perennial herb	Dried rhizome	Carminative
Matum (มะตม)	*Aegle marmelos* (L.) Corrêa	Rutaceae	Tree	Fruits and bark	Antidiarrheal, stomachic
Hom (หอม)	*Allium ascalonicum* L.	Amaryllidaceae	Biennial herb	Dried bulb	Carminative, expectorant
Krathiam (กระเทยม)	*Allium sativum* L.	Amaryllidaceae	Herb	Bulb	Antimicrobial, antihyperlipidemic
Fa Thalai (ฟ้าทะลายโจร)	*Andrographis paniculata* (Burm. f.) Nees	Acanthaceae	Herb	Dried aerial part	Antidiarrheal, antipyretic, antiinflammatory
Thian Ta Takkatan (เทยนตาตกแตน)	*Anethum graveolens* L.	Apiaceae	Annual herb	Dried ripe fruit	Carminative, pharmaceutic aid
Kot (โกฐสอ)	*Angelica dahurica* (Hoffm.) Benth. & H	Apiaceae	Perennial herb	Dried root	Antipyretic, analgesic
Kot Chiang (โกฐเชยง)	*Angelica sinensis* (Oliv.) Diels	Apiaceae	Perennial herb	Roots	Blood tonic, treatment of mental disorders
Khamin Khruea (ขม้นเครอ)	*Arcangelisia flava* (L.) Merr.	Menispermaceae	Large climber	Stem	Stomachic, antidiarrheal, antibacterial
Maksong (หมากสง)	*Areca catechu* L.	Areceae	Small or medium sized tree	Ripe seed	Anthelmintic, antidiarrheal,
Kot Chula Lampha (โกฐจฬาลมพา)	*Artemisia annua* L.	Asteraceae	Annual herb	Dried aerial part	Antipyretic
Kot Khamao (โกฐเขมา)	*Atractylodes lancea* (Thunb.) DC.	Asteraceae	Perennial herb	Rhizome	Stomachic
Kot Kraduk (โกฐกระดก)	*Aucklandia lappa* Decne	Asteraceae	Herb	Roots	Stomachic, carminative, antispasmodic
Sawat (สวาด)	*Caesalpinia bonduc* (L.) H. Roxb.	Fabaceae	Climber	Leaf	Laxative, antiflatulent
Phrik Khinu (พรกขหน)	*Capsicum annuum* L.	Solanaceae	Annual or perennial herb	Dried ripe fruit	Gastro-intestinal stimulant, counterirritant
Thian Ta Kop (เทยนตากบ)	*Carum carvi* L.	Apiaceae	Perennial herb	Dried ripe fruit	Carminative, antiflatulent, pharmaceutic aid
Khun (คน)	*Cassia fistula* L.	Fabaceae	Tree	Pulp	Laxative
Buabok (บวบก)	*Centella asiatica* (L.) Urb.	Apiaceae	Herb	Aerial part	Mild diuretic, antiinflammatory, wound healing
Phet Sangkhat (เพชรสงฆาต)	*Cissus quadrangularis* L.	Vitaceae	Woody climber	Dried stem	Alleviation of hemorrhoidal symptoms
Makrut (มะกรด)	*Citrus hystrix* DC.	Rutaceae	Shrub or small tree	Leaf	Pharmaceutics aid, carminative
Phaya Yo (พญายอ)	*Clinacanthus nutans* (Burm. f.) Lindau	Acanthaceae	Scandent shrub	Leaf	Antiinflammatory, antiviral
Thian Khao (เทยนขาว)	*Cuminum cyminum* L.	Apiaceae	Herb	Fruit	Carminative, expectorant, alterative
Khamin Chan (ขม้นชน)	*Curcuma longa* L.	Zingiberaceae	Perennial herb	Dried rhizome	Stomachic, carminative, pharmaceutics aid, astringent
Khamin Oi (ขม้นออย)	*Curcuma* sp.	Zingiberaceae	Perennial herb	Dried rhizome	Stomachic, antidiarrheal, emmenagogue
Lakkachan (ลกจน)	*Dracaena cochinchinensis* (Lour.) S. C. Chen	Asparagaceae	Tree	Wood	Antipyretic, antiinflammatory
Pla Lai Phueak (ปลาไหลเผอก)	*Eurycoma longifolia* Jack	Simaroubaceae	Shrub	Roots	Antipyretic
Thian Khao Plueak (เทยนขาวเปลอก)	*Foeniculum vulgare* Mill.	Apiaceae	Herb	Dried cremocarp and mericarp	Carminative, spasmolytic
Krachiap Daeng (กระเจยบแดง)	*Hibiscus sabdariffa* L.	Malvaceae	Annual herb or subshrub	Dried persistent calyx and epicalyx	Diuretic
Maenglak Kha (แมงลกคา)	*Hyptis suaveolens* (L.) Poit.	Lamiaceae	Shrub	Dried aerial part	Carminative, antimicrobial (topical)
Krachai Dam (กระชายดำ)	*Kaempferia parviflora* Wall. ex Baker.	Zingiberaceae	Herb	Rhizome	Tonic, carminative
Thian Daeng (เทยนแดง)	*Lepidium sativum* L.	Brassicaceae	Annual herb	Seeds	Expectorant, stomachic
Kot Hua Bua (โกฐหวบว)	*Ligusticum sinense* Oliv. cv. Chuanxiong	Apiaceae	Herb	Dried Rhizome	Carminative, blood tonic for menstrual disorder
Bunnak (บนนาค)	*Mesua ferrea* L.	Calophyllaceae	Tree	Dried blooming flower	Cardiotonic, antipyretic
Phikun (พกล)	*Mimusops elengi* L.	Sapotaceae	Tree	Dried flower	Tonic, antipyretic
Mara Khi Nok (มะระข้นก)	*Momordica charantia* L.	Cucurbitaceae	Annual or perennial climber	Fruit	Bitter tonic, internal heat alleviating
Mon (หมอน)	*Morus alba* L.	Moraceae	Deciduous tree or shrub	Leaf	Mild antitussive
Kot Chada Mangsi (โกฐชฎามงส)	*Nardostachys jatamansi* (D. Don) DC.	Caprifoliaceae	herb	Roots, Rhizome	Mild sedative, treatment of dysmenorrhea
Bua Luang (บวหลวง)	*Nelumbo nucifera* Gaertn.	Nelumbonaceae	Perennial herb	Dried stamen	Cardiotonic, antipyretic
Kot Kan Phrao (โกฐก้านพร้าว)	*Neopicrorhiza scrophularia* Pennell	Plantaginaceae	Perennial herb	Rhizome	Antipyretic, stomachic
Thian Dam (เทยนดำ)	*Nigella sativa* L.	Ranunculaceae	Annual herb	Seed	Carminative, diuretic
Kaphrao Daeng (กะเพราแดง)	*Ocimum tenuiflorum* L.	Lamiaceae	Herb	Dried leaf	Pharmaceutics aid, carminative
Ya Nuat Maeo (หญ้าหนวดแมว)	*Orthosiphon aristatus* (Blume) Miq.	Lamiaceae	Perennial herb	Dried leaf and stem tip	Diuretic
Makham Pom (มะขามปอม)	*Phyllanthus emblica* L.	Phyllanthaceae	Small or medium sized tree	Dried mature fruit	Expectorant, laxative, antiscorbutic
Thian Sattabut (เทยนสตตบษย)	*Pimpinella anisum* L.	Apiaceae	Annual herb	Dried ripe fruit	Carminative, expectorant, pharmaceutic aid
Phlu (พล)	*Piper betle* L.	Piperaceae	Woody climber	Leaf	Antifungal, antiallergic
Phrik Thai Dam (พรกไทยดำ)	*Piper nigrum* L.	Piperaceae	Climber	fruit	Aromatic, stomachic, carminative
Phrik Thai Lon (พรกไทยลอน)	*Piper nigrum* L.	Piperaceae	Woody perennial climber	Dried unripe fruit	Aromatic, stomachic, carminative
Di Pli (ดปล)	*Piper retrofractum* Vahl	Piperaceae	Woody climber	Dried stem	Carminative, stomachic, antiinflammatory
Chaphlu (ชะพล)	*Piper sarmentosum* Roxb.	Piperaceae	Herb	Leaf	Carminative
Sakhan (สะค้าน)	*Piper wallichii* (Miq.) Hand.-Mazz.	Piperaceae	Woody climber	Dried stem	Carminative, stomachic, antiinflammatory
Thian Klet Hoi (เทยนเกลดหอย)	*Plantago ovata* Forssk.	Plantaginaceae	Herb	Seed	Bulk-forming laxative
Chan Daeng (จนทนแดง)	*Pterocarpus santalinus* L. f.	Fabaceae	Tree	Bark	Antipyretic, antiinflammatory, cardiotonic
Chan Khao (จนทนขาว)	*Santalum album* L.	Santalaceae	Tree	Dried heartwood	Cardiotonic, stomachic, nerve tonic
Chumhet Thet (ชมเหดเทศ)	*Senna alata* (L.) Roxb.	Fabaceae	Herb or under shrub	Dried mature seed	Laxative, antifungal
Chumhet Thai (ชมเหดไทย)	*Senna tora* (L.) Roxb.	Fabaceae	Herb or under shrub	Dried mature seed	Laxative, diuretic
Mawaeng Khruea (มะแวงเครอ)	*Solanum trilobatum* L.	Solanaceae	Slender scrambling shrub	Fruit	Expectorant
Thaowan Priang (เถาวลยเปรยง)	*Solori scandens* (Roxb.) Sirich. & Ade	Fabaceae	Large woody Climber	Dried stem	Analgesic, antiinflammatory,
Tanmon (ตานหมอน)	*Tarlmounia elliptica* (DC.) H. Rob., S. C.	Asteraceae	Scandent shrub	Leaf	Demulcent
Samo Phiphek (สมอพเภก)	*Terminalia bellirica* (Gaertn.) Roxb.	Combretaceae	Large tree	Mature fruit	Laxative, carminative, astringent, expectorant
Samo Thai, Kot Phung Pla (สมอไทย, โกฐพงปลา)	*Terminalia chebula* Retz.	Combretaceae	Large tree	Mature fruit	Laxative, carminative, astringent, expectorant
Rangchuet (รางจด)	*Thunbergia laurifolia* Lindl.	Acanthaceae	Woody climber	Leaf	Detoxicant, antipyretic
Boraphet (บอระเพด)	*Tinospora crispa* (L.) Hook. f. & Thom	Menispermaceae	Woody climber	Dried stem	Antipyretic, bitter tonic, stomachic
Thian Yaowaphani (เทยนเยาวพาณ)	*Trachyspermum ammi* (L.) Sprague	Apiaceae	Annual herb	Dried ripe fruit	Carminative, pharmaceutic aid
Phlai (ไพล)	*Zingiber montanum* (J. König) Link. ex A.	Zingiberaceae	Herb	Dried rhizome	Antiinflammatory, counterirritant, mosquito repellent
Khing (ขง)	*Zingiber officinale* Roscoe	Zingiberaceae	Perennial herb	Dried rhizome	Carminative, antiflatulent
Krathue (กระทอ)	*Zingiber zerumbet* (L.) Sm.	Zingiberaceae	Herb	Dried rhizome	Antiflatulent, stomachic

**Table 2:** Species admixtures in the herbal trade samples of medicinal plants listed in the Thai Herbal Pharmacopoeia and techniques employed for discrimination.

Thai name	Scientific name	Matrix type	Total number samples	Percentage of species admixture detected	Declared/identified species	Discriminant technique employed	Reference
Maksong (หมากสง)	*Areca catechu* L.	Processed sample	45	38.09	Nil	Mini-DNA barcode	Song et al., 2017
Matum (มะตม)	*Aegle maamelos*, (L.) Corrêa	Leaf, root, fruit	11	0	Nil	DNA barcode	Kumar et al., 2018
Fa Thalai (ฟ้าทะลาย)	*Andrographis paniculata* (Burm.f.) Nees	Dried sample, powder, capsule, tea	10	NQ	*Andrographis paniculata, Acanthus ebracteatus* and *Rhinacanthus nasutus*	DNA barcode	Osathanunkul et al., 2016
Thian Ta Takkatan (เทยนตาตกแตน)	*Anethum graveolens* L.	N/A	N/A	NQ	*Trachyspermum ammi andFoeniculum vulgare*	DNA barcode	Schori and Showalter, 2001
Kot (โกฐสอ)	*Angelica dahurica* (Hoffm.) Benth. & Hook.f. ex Franch. & Sav.	Root	N/A	NQ	Nil	Metabarcoding and real-time PCR	Xin et al., 2018
Kot (โกฐสอ)	*Angelica dahurica* (Hoffm.) Benth. & Hook.f. ex Franch. & Sav.	Root	20	NQ	*A. anomala* and *A. japonica*	DNA barcode and SCAR assay	Noh et al., 2018
Kot Chiang (โกฐเชยง)	*Angelica* sinensis (Oliv.) Diels	N/A	N/A	NQ	*A. laxifoliata* and *A. nitida*	DNA barcode	Feng et al., 2010
Kot Chiang (โกฐเชยง)	*Angelica* sinensis (Oliv.) Diels	Root	13	NQ	Nil	HPLC fingerprints	Lu et al., 2005
Kot Chula Lampha (โกฐจฬาลมพา)	*Artemisia annua* Pall.	Dried herb, powder, tablet, tea	58	NQ	*A. atrovirens* and *A. indica*	High resolution melting (HRM) curve analysis and DNA barcode	Song et al., 2016
Phrik Khinu (พรกข้หน)	*Capsicum annuum* L.	Powder	5	NQ	*Beta vulgaris* and *Ziziphus nummularia*	RAPD-PCR and SCAR markers	Dhanya et al., 2011
Phrik Khinu (พรกข้หน)	*Capsicum annuum* L.	Powder	61	14.75	*Garlic, spring onion*, and/or *onion*	Real-time PCR and DNA sequencing	Kang, 2018
Khun (คน)	*Cassia fistula* L.	Dried herb, powder,	12	0	Nil	DNA barcode	Seethapathy et al., 2015
Thian Khao, Yira (เทยนขาว/ยหร)	*Cuminum cyminum* L.	Powder	11	NQ	Nil	DNA barcode	Arunraj et al., 2016
Khamin Chan (ขม้นชน)	*Curcuma longa* L.	Dried and fresh plant tissue	7	58.54	Nil	Bar-HRM technique	Osathanunkul et al., 2017
Pla Lai Phueak (ปลาไหลเผอก)	*Eurycoma longifolia* Jack	Root powder	46	50 or more	Nil	HPLC and two-dimensional electrophoresis (2DE)	Vejayan et al., 2018
Pla Lai Phueak (ปลาไหลเผอก)	*Eurycoma longifolia* Jack	Capsule, tablet, tea	11	27	*Holcoglossum* sp., *Nigella arvensis, Nigella sativa,* and *Ficus deltoidea*	DNA barcode and HPLC analysis	Abubakar et al., 2018
Pla Lai Phueak (ปลาไหลเผอก)	*Eurycoma longifolia* Jack	Capsule, beverage, instant coffee mix, tea	11	NQ	Nil	Bar-HRM analysis	Fadzil et al., 2018
Krachai Dam, Thai Ginseng (กระชายดำ)	*Kaempferia parviflora* Wall. ex Baker	Processed and packed commercial powder	7	58.54	Nil	Bar-HRM analysis	Osathanunkul et al., 2017
Bunnak (บนนาค)	*Mesua ferrea* L.	Crude drug	6	33	Nil	DNA barcode	Kumar et al., 2018
Thian Dam (เทยนดำ)	*Nigella sativa* L.	Seed	10	NQ	*Allium cepa* and *Clitoria guianensis*	DNA barcode	Sudhir et al., 2016
Thian Dam (เทยนดำ)	*Nigella sativa* L.	Seed oil	N/A	NQ	Grape seed oil	Fourier transform infrared (FTIR) spectroscopy and gas chromatography	Nurrulhidayah et al., 2011
Phrik Thai (พรกไทย)	*Piper nigrum* L.	Fruit	N/A	NQ	*Carica papaya*	HPLC and antioxidative assay markers	Menghani et al., 2010
Phrik Thai (พรกไทย)	*Piper nigrum* L.	Powder	9	NQ	Chili	DNA barcode and HPLC	Parvathy et al., 2014
Phrik Thai (พรกไทย)	*Piper nigrum* L.	Seed	N/A	NQ	*Carica papaya*	RAPD markers	Khan et al., 2010
Chan Khao (จนทนขาว)	*Santalum album* L.	Oil	38	NQ	Nil	Gas chromatography– mass spectrometry	Howes et al., 2004
Chan Khao (จนทนขาว)	*Santalum album* L.	Oil	6	NQ	Nil	Multidimensional gas chromatography with simultaneous mass spectrometric and flame ionization detection	Sciarrone et al., 2011
Samo phiphek (สมอพเภก)	*Terminalia billirica* (Gaertn.) Roxb.	Fruit	10	0	Nil	DNA barcode	Kumar et al., 2018
Samo phiphek (สมอพเภก)	*Terminalia bellirica (Gaertn.) Roxb.*	Crude drug	12	NQ	Nil	PCR-RFLP and amplification refractory mutation system (ARMS)	Intharuksa et al., 2016
Samo Thai (สมอไทย)	*Terminalia chubula* Retz.	Fruit	13	0	Nil	DNA barcode	Kumar et al., 2018
Samo Thai (สมอไทย)	*Terminalia chebula* Retz.	Immature fruit	N/A	NQ	Nil	Chromatographic fingerprint analysis	Xie et al., 2006
Rangchuet (รางจด)	*Thunbergia laurifolia* Lindl.	Leaf	8	NQ	Nil	PCR-RFLP	Suwanchaikasem et al., 2013
Rangchuet (รางจด)	*Thunbergia laurifolia* Lindl.	Both fresh and dried sample, powder	10	NQ	Nil	Bar-HRM analysis	Singtonat and Osathanunkul, 2015

A number of studies have reported species admixtures in herbal trade across countries. For example, DNA-based approaches have been used for the identification of species listed in Amazonian traditional medicine (Mezzasalma et al., 2017). *Trachelospermum jasminoides* is commonly used as traditional Chinese medicine and sold in markets in dried and sliced forms, which pose difficulties in traditional identification methods. Yu et al. (2017) used the nuclear region ITS2 to evaluate the 127 sequences representing *T. jasminoides via* the neighbor-joining tree method, which demonstrated the remarkable use of DNA barcoding to authenticate market samples. Using the ITS2 region, Chen et al. (2017) found that only 78% of the market samples contained the species listed on their product label. A few studies showed the use of both molecular and chemical markers and phylogenetic approaches for the identification herbal products, including dietary supplements. For example, a study using a phylogenomic approach analyzed the evolutionarily complex genus *Berberis* in order to develop DNA barcodes for the medicinally important species *Berberis aristata* for the regulatory purposes and quality control (Kreuzer et al., 2019). The combination of molecular and chemical markers ensures the quality of the Copalchi complex used in Mexican Herbal Pharmacopoeia and successfully differentiates between the species *Hintonia latiflora*, *H. standleyana*, and *Exostema caribaeum* (Cristians et al., 2018). Pawar et al. (2017) used traditional DNA barcoding techniques and chemical markers to identify frequently consumed botanical dietary supplements of ginkgo, soy, valerian, yohimbe, etc. The combination of DNA barcoding and nuclear magnetic resonance (NMR) was used for the identification of admixture in *Garciania* species (Seethapathy et al., 2018) and *Saraca acosa* (Urumarudappa et al., 2016). Wang et al. (2016) used DNA barcoding for the identification of processed *Angelicae sinensis* radix (Danggui) used in Chinese patent medicines (CPMs). *Lonicerae japonicae* Flos was used to produce hundred kinds of CPMs, single nucleotide polymorphisms (SNPs) were used to generate mixtures of powdered CPMs for authentication, and other CPMs were generated through substitutions or as adulterants (Gao et al., 2017). Zhang et al. (2017) used SNPs for which traditional DNA barcoding has not been successful for the differentiation of *Echinacea* species.

Loop-mediated isothermal ampliﬁcation (LAMP) is one of the approaches developed to identify herbal medicine species (Li et al., 2016). A study demonstrated that the recombinase polymerase amplification (RPA) assay can be developed into an efficient tool for the rapid on-site authentication of plant species in *Ginkgo biloba* herbal products to differentiate the two species *G. biloba* and *Sophora japonica* (as adulteration) (Liu et al., 2018). DNA barcoding and metabarcoding have potential for the quality control of herbal products (Raclariu et al., 2018). Recently, a study recounted the history of DNA-based methods for identification of botanicals, discussed some of the difficulties in defining a specific barcode or codes to use, and described how next generation sequencing technologies have enabled new techniques that can be used to identify these products with great authority and resolution (Moraes et al., 2015). High-throughput sequencing (HTS) methods were used effectively in the quality control and identification of food components. The HTS platforms, Illumina, and Ion Torrent, were used for the analysis of herbal teas, which yielded congruent results, both qualitatively and quantitatively (Speranskaya et al., 2018). These studies confirm that species admixtures may occur in the raw herbal trade. An intrinsic problem associated with the adulteration of herbal products is the effect it may have on user health and safety (Valiathan, 2006; Seethapathy et al., 2015; Srirama et al., 2017). Without regulation, such adulteration will decrease the efficacy and consumer confidence in herbal products, which will eventually cause economic damage to the raw herbal trade (Srirama et al., 2017).

### Policies and Regulations Concerning Herbal Medicines

Recently, the large-scale international trade of herbal products has increased the concern for the safety and efficacy of herbal products (Walker and Applequist, 2012; Newmaster et al., 2013; Techen et al., 2014; Srirama et al., 2017). Since most herbal medicines are used in crude formulas in combinations of several herbs and often have extended usage, it is important that the species being used undergo strict validation, safety assessment, and quality and regulatory approval, similarly to modern medicinal drugs (Srirama et al., 2017). With continued development and improvements in DNA barcoding technology, especially with the combination of high-throughput techniques developed for DNA barcoding, a large number of samples can be assessed (Srirama et al., 2017). This technology could ensure the validation of raw herbal products and the identification of a large number of species *via* DNA-based methods (Srirama et al., 2017).

Although herbal medicines are widely used in healthcare systems for the treatment, diagnosis, and control of disease, quality control and proper regulation remain the foremost challenges worldwide. Every nation has its own official compendiums detailing the standardization and quality procedures for traditional medicine production. Although the WHO has passed stringent regulations related to traditional product production and formulation, very few countries have implemented regulations for herbal medicines, and most countries do not have proper guidelines for botanicals. Therefore, the quality of traded medicinal herbal products is not guaranteed (WHO, 2017).

Thailand became the 26th member of the WHO in 1984, which was a year after the Adverse Drug Reaction Monitoring Center (ADRMC) was associated under the authority of the Thai Food and Drug Administration (Thai FDA) (WHO, 2017). The Thai FDA collects reports from health product surveillance systems and programs through a database, Thai herbal database. The Thai herbal database is a potentially effective data source for identifying adverse events related to herbal products (Saokaew et al., 2011). This database provides specific data with respect to the adverse status of a particular plant species (http://thaihpvc.fda.moph.go.th/thaihvc/index.jsf#). However, there is an immediate need for a quality check portal before it reaches the consumer.

### Quality Control: Centralized Testing Laboratory Proposition

Natural products are gaining popularity each day due to their safety and availability at an affordable price. The words “herbal,” “natural,” and “plant-derived” can be misleading at times, and it is important for the public to be made aware that herbal mixtures are medicines in their own right (Fan et al., 2012). Herbal medicinal preparations are formulations commonly consisting of 5 to 15 different herbs or a complex formulation consisting of several medicinal herbs and chemical drug constituents. A single herbal medicine may contain many natural constituents and/or a combination of numerous herbs that can give rise to interactions with hundreds of natural ingredients (Choi et al., 2002). This demands multiple stages of assessment of herbal extracts or products, such as pharmaceutical documentation, toxicology studies, and clinical studies for quality, safety, and efficacy, before entering the market.

**Figure 1:**
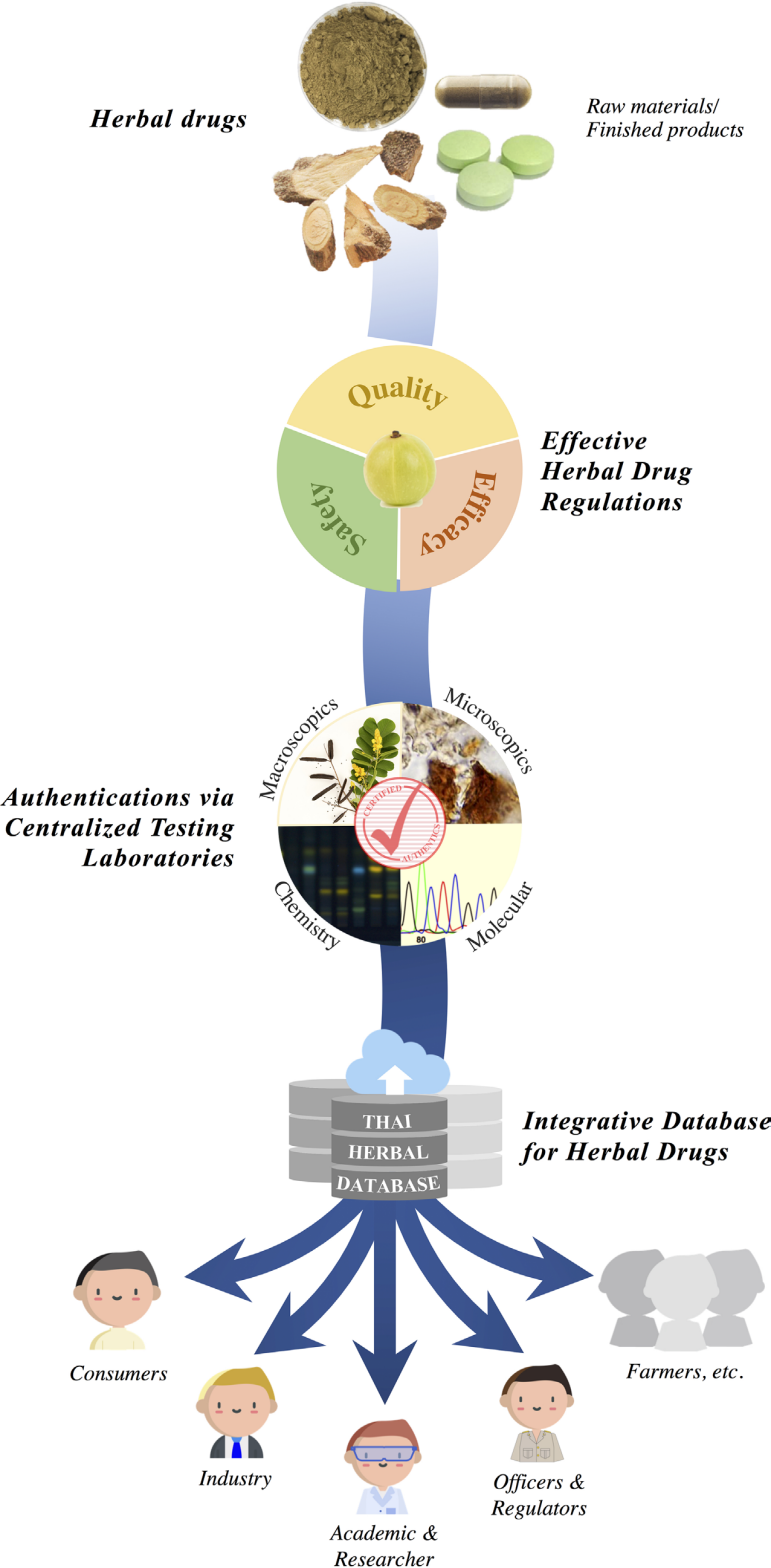
Proposed regulatory framework of traded herbal drugs of Thailand.

A strong regulatory mechanism must be implemented to screen the safety, identity, and quality of herbal products (Srirama et al., 2017). Central to such a regulatory structure is the establishment of an effective entity to evaluate the credibility of species, assess the authenticity of the raw herbal products, and connect these data with trade regulators, both nationally and internationally. To safeguard the integrity of herbal trade, trade partners may need to access and utilize a biological reference guide developed through molecular diagnostic tools coupled with metabolite profiling. Ensuring herbal medicine quality also makes the products safer and more reliable, efficacious, sustainable, and marketable (Srirama et al., 2017). Imports and exports can be governed by such validations certified by nationally recognized government bodies/agencies (**Figure 1**). It is important that such a governing body also sets up a regulatory system wherein both traditional medicinal plants and their herbal products are placed. Furthermore, it is important that the molecular tools (DNA sequences) and chemical metabolite profiles are also available along with the herbal plant samples (Srirama et al., 2017). These tools would provide a simple reference for validation. However, Thai medicinal products containing mixtures of numerous herbs make it difficult, time consuming, and expensive to meet the requirements of identification (both by DNA-based and metabolite-based methodologies). However, with the emergence of new technologies, especially next-generation and high-throughput techniques, a large number of samples can be evaluated through meta-barcoding and chemical analysis. In addition to the potential for frequent authentication of herbal products, this setup can also identify a wide variety of medicinal plant species.

**Figure 2:**
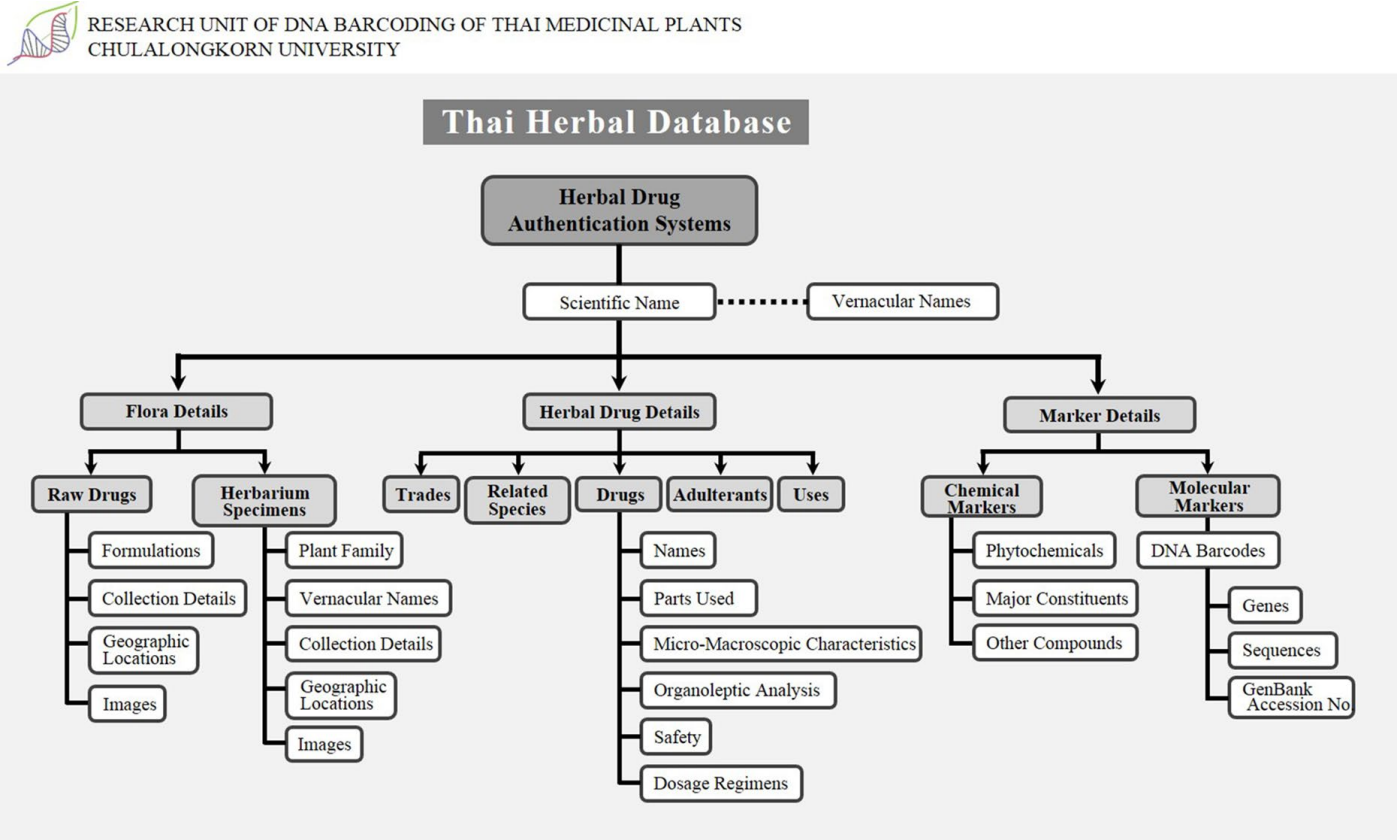
Schematic representation of the Thai Herbal database.

Safe and stable herbal extracts may be marketed if their therapeutic use is well documented and certified by a CT laboratory. There is an urgent need to develop a digital key that would enable easy identification of medicinal plants and their products, which could integrate floristic details, trade, drug databases, and DNA barcoding information (**Figure 2**). Electronic access to such database information can be made possible with internet access and international data sharing efforts, where a wealth of chemical and DNA sequence information can be made available and samples be compared. Users can easily search and compare by plant name or drug name to obtain complete details.

### Conclusions

Medicinal plants and their products are extensively used in the indigenous and modern healthcare systems in Thailand and many countries. The international trade of herbal products has a major impact on the international economy, and the demand for herbal products is growing in both developing and developed countries. This increase in demand has resulted in the substitution and adulteration of medicinal plants with other herbal products whose health benefits are unknown. The results of this study would potentially be useful in providing recommendations to address the increasing concerns of adulteration in raw drug trade and to propose mechanisms that can ensure quality standards in raw drug markets.

Our observations clearly support the claim that there is no established mechanism to connect and coordinate the herbal industry for the certification of herbal products in Thailand. Therefore, we propose the Thai herbal database that contains data on all medicinal plants, including floristic details such as taxonomic hierarchy, vernacular names in various languages, habitat, cultivation type, worldwide distribution maps, Thailand distribution maps (state and district), species images, herbarium images, general photographs, line diagrams, and synonyms. Genetic data include DNA marker information, gene sequences, GenBank accession number details, and chemical data include details of various kinds of tests such as TLC identity test, gas chromatography, gas liquid chromatography, and estimation of chemical compounds in drugs using HPLC and a complete list of major chemical constituents (marker compounds). It also contains a list of other chemical constituents and possibly important chemical compound structures as well as the status of import–export trade and the details of admixtures found in those particular plants. This database could play an important role in monitoring the medicinal plant trade and could be a promising initiative in Thailand for the development of science and technology and to provide consumers with access to all essential information. This herbal database concept could be a novel strategy in Thailand, generating transparency for all safety and quality measures and facilitating the prevention of admixtures in the herbal trade. This herbal database should be developed with utmost planning and made available to all researchers, academicians, people involved with regulatory policy and industry, and, most importantly, common people so that they may gain access to past and present studies. Additionally, in our opinion, accessibility of this herbal database to all researchers, traders, and consumers will allow the sensible development of drug safety measures. The use of this concept can allow governing bodies to improve the efficacy of herbal drugs at a considerable cost.

## Author Contributions

Conceptualization and design of the study: SS and SK. Data collection and formal analysis: SK. Validation and visualization: SK and CT. Conclusion: SS. Manuscript writing: SK. Review and editing: SS and SK.

## Funding

The Ratchadaphisek Somphot Endowment Fund, Graduate School, Chulalongkorn University provided funding which was used to assist in the preparation of this review.

## Conflict of Interest

The authors declare that the research was conducted in the absence of any commercial or financial relationships that could be construed as a potential conflict of interest.
